# A Systems-Level Approach to Studying Birth Defects: Novel Method Identifies Potential Key Pathway

**DOI:** 10.1289/ehp.121-a95

**Published:** 2013-03-01

**Authors:** Kellyn S. Betts

**Affiliations:** Kellyn S. Betts writes about environmental contaminants, hazards, and technology for solving environmental problems for publications including *EHP* and *Environmental Science & Technology*.

Birth defects are a leading cause of infant mortality, and the majority of defects have unknown causes. Now researchers at the University of North Carolina–Chapel Hill have identified the glucocorticoid receptor pathway as a key mediator of birth defects caused by exposure to inorganic arsenic [*EHP 121(3):332–338; Ahir et al.*]. The researchers used a three-part strategy to test their hypothesis that a systems-level approach could uncover biological pathways involved in metal-induced birth defects.

The researchers were inspired to begin the project as a result of their ongoing studies to identify environmental causes of birth defects in North Carolina. They focused on seven metals commonly found in food, drinking water, air, and/or consumer products. All the metals are known or suspected to be developmental toxicants.

The first step was to search the publicly available Comparative Toxicogenomics Database for genes with any known relationship to at least one of the seven metals as well as to developmental defects. At the time of the study the database contained over 178,000 interactions between nearly 5,000 chemicals and more than 16,000 genes and proteins in 298 species. (The database has since grown significantly.)

Next, the researchers used systems-level computer analyses to overlay the identified genes onto known molecular networks to see which biological pathways were represented. They pinpointed the glucocorticoid receptor pathway as being significantly associated both with development and with exposure to arsenic, cadmium, mercury, and selenium.

**Figure:**
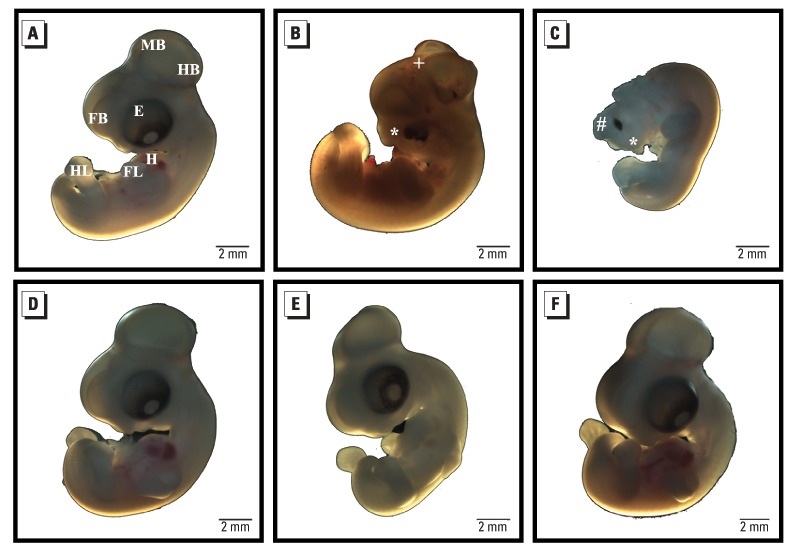
Six-day-old embryos from each treatment group were treated with inorganic arsenic or with phenytoin, a pharmaceutical known to induce birth defects. (A) A control embryo shows normal development of the forebrain (FB), midbrain (MB), hindbrain (HB), eye (E), heart (H), fore limb buds (FL), and hind limb buds (HL). (B) A phenytoin-treated embryo shows an abnormal head shape, failure of the neural tube to close properly (+), and craniofacial defects (*). (C) An arsenic-treated embryo shows craniofacial defects (*) and anterior neural tube defects (#). When the glucocorticoid receptor pathway was blocked in controls (D), phenytoin-treated embryos (E), and arsenic-treated embryos (F), such defects did not occur. (Adapted from Ahir et al., doi:10.1289/ehp.1205659.)

The final step was to test the bioinformatically generated predictions in the laboratory setting. The team used *in ovo* chick embryos, an established model for assessing teratogenicity, to evaluate the effects of exposure to inorganic arsenic. They found that levels of arsenic as low as 7.5 ppb induced structural defects including microcephaly, neural tube defects, and gross craniofacial defects. When the glucocorticoid receptor pathway was blocked, chick embryos did not develop structural defects when exposed to arsenic.

Although the glucocorticoid receptor has been studied in relation to health effects of metal exposures, relatively little attention has been paid to its potential role in birth defects. One implication of this research—and a major strength of the study—is the ability to cost-effectively identify candidate biological pathways for further study in association with environmentally induced birth defects.

